# Postoperative Pain, Swelling, and Trismus After Piezosurgery-Assisted Minor Oral Surgery in Patients with Coronary Artery Disease and Diabetes Mellitus: A Retrospective Cohort Study

**DOI:** 10.3390/jcm15155874

**Published:** 2026-07-27

**Authors:** Mert Zeytinoğlu, Meltem Özden Yüce, Mehmet Cemal Akay

**Affiliations:** Department of Oral and Maxillofacial Surgery, School of Dentistry, Ege University, 35040 Izmir, Turkey; meltemozdn@hotmail.com (M.Ö.Y.); cemalakay@yahoo.com (M.C.A.)

**Keywords:** piezosurgery, coronary artery disease, diabetes mellitus, medically compromised patients, postoperative outcomes

## Abstract

**Background/Objectives:** This retrospective cohort study aimed to evaluate postoperative pain, swelling, and trismus following piezosurgery-assisted minor oral surgical procedures in patients with coronary artery disease and diabetes mellitus (CAD+DM) and to compare the outcomes with those of systemically healthy individuals. **Methods:** Medical records of 60 patients who underwent piezosurgery-assisted minor oral surgery between 2018 and 2021 were retrospectively reviewed. Patients were allocated into two groups: CAD+DM (n = 30) and healthy controls (n = 30). Postoperative pain was assessed using a visual analog scale (VAS) at 6 h, 12 h, postoperative day 1, day 2, day 3, and day 7. Swelling and trismus were evaluated preoperatively and on postoperative days 2 and 7. Swelling and trismus data were analyzed using repeated-measures ANOVA and two-way repeated-measures ANOVA, whereas VAS scores were analyzed using the Friedman test, Dunn’s post hoc test, and Mann–Whitney U test. **Results:** Both groups demonstrated significant time-dependent changes in swelling and trismus (all *p* < 0.001). Swelling increased on postoperative day 2 and decreased by day 7 in both groups; however, swelling values were significantly higher in the CAD+DM group at all evaluation time points (all *p* < 0.0001). Trismus was greatest on postoperative day 2 and improved significantly by day 7 in both groups (all *p* < 0.0001). A significant time × group interaction was observed for trismus (*p* < 0.0001), whereas no significant between-group difference was detected on postoperative day 7 (*p* = 0.7729). VAS scores decreased significantly over time in both groups (Friedman test, *p* < 0.0001). Pain scores were significantly lower in the CAD+DM group at 6 h, day 1, day 2, day 3, and day 7 (all *p* < 0.001), while no difference was observed at 12 h (*p* = 0.633). **Conclusions:** Piezosurgery-assisted minor oral surgery was associated with favorable postoperative recovery in both CAD+DM and healthy patients. Although CAD+DM patients exhibited greater postoperative swelling, trismus recovery was comparable between groups, and postoperative pain remained low throughout follow-up. Piezosurgery appears to be a safe and effective surgical approach for medically compromised patients undergoing minor oral surgical procedures.

## 1. Introduction

Piezoelectric bone surgery has become an established technique in oral and maxillofacial surgery because ultrasonic microvibrations enable selective cutting of mineralized tissues while largely preserving adjacent soft tissues, vessels, and nerves [1,2]. Compared with conventional rotary instruments, piezosurgery offers micrometric osteotomy, improved intraoperative visibility through its cavitation effect, and reduced mechanical and thermal trauma to the surgical field [1]. These properties have supported its use in a broad range of procedures, including impacted tooth surgery, alveoloplasty, ridge modification, sinus floor elevation, implant-site preparation, and other maxillofacial interventions [1,3]. In routine oral surgery, the main clinical value of piezosurgery lies not only in precise bone cutting but also in its potential to reduce postoperative morbidity, particularly pain, facial swelling, and trismus [2,3]. However, the clinical evidence base supporting these advantages has been derived predominantly from third-molar or otherwise systemically healthy cohorts, limiting direct extrapolation to medically vulnerable patients with substantial vascular and metabolic comorbidities [2,3].

Postoperative morbidity remains a major determinant of patient comfort and early functional recovery after minor oral surgery, especially in procedures requiring flap elevation and bone removal [2,3]. Previous clinical studies have shown that piezosurgery may be associated with a more favorable postoperative course than conventional burs, with less soft-tissue trauma and less pronounced inflammatory sequelae during early healing [2,3]. For medically compromised patients, even modest differences in early postoperative swelling, pain, or mouth-opening limitation may have greater implications for oral function and short-term recovery than in younger, systemically healthy individuals [4,5]. As a result, the external validity of findings from healthy populations remains uncertain when applied to patients with major systemic disease [4,5]. Accordingly, the relevant clinical question is not only whether piezosurgery improves recovery in general, but also whether its tissue-sparing profile may be particularly advantageous in patients whose healing biology is already compromised [5,6].

This issue is clinically important because coronary artery disease and diabetes mellitus are among the most prevalent chronic disorders worldwide and commonly coexist [7,8]. Cardiovascular diseases remain the leading cause of death globally, accounting for an estimated 19.8 million deaths in 2022, with coronary heart disease constituting a major component of this burden [7]. In parallel, the 11th edition of the IDF Diabetes Atlas reported that 589 million adults aged 20–79 years were living with diabetes in 2025, and this number is projected to rise to 853 million by 2050 [8]. The overlap between cardiovascular disease and diabetes is particularly relevant in oral surgery because these patients frequently present with cumulative vascular and metabolic impairment, which may influence perioperative tissue response and postoperative recovery [5,7].

Diabetes mellitus may further complicate postoperative recovery because chronic hyperglycemia is linked to endothelial dysfunction, microvascular impairment, oxidative stress, advanced glycation end-product accumulation, altered inflammatory signaling, delayed collagen remodeling, and impaired wound healing [4,5]. Contemporary wound-healing research emphasizes that diabetic tissue repair is marked not only by delayed angiogenic and fibroproliferative responses but also by dysregulated macrophage activity, persistent low-grade inflammation, and defective re-epithelialization [5]. In addition, diabetes-related peripheral neuropathy can modify nociception and may alter how postoperative pain is perceived and reported [6]. This is particularly relevant when postoperative pain is used as a surrogate for surgical recovery, because sensory dysfunction may reduce symptom reporting without fully reflecting the magnitude of the underlying inflammatory response [6]. These mechanisms are clinically relevant in oral surgery because they can influence not only tissue healing but also the severity and duration of swelling, trismus, and pain after intervention [4,5,6]. Therefore, a surgical approach that minimizes collateral soft-tissue injury may be especially advantageous in patients with combined cardiovascular and metabolic disease [1,5].

Despite this rationale, clinical data on piezosurgery in medically compromised patients remain scarce, and evidence specifically comparing patients with coronary artery disease and diabetes mellitus to healthy controls is limited [2,3]. Most studies have emphasized technical feasibility or comparisons of cutting instruments, whereas fewer have examined how systemic disease status may influence postoperative outcomes after piezoelectric surgery [2,3]. As a result, an important knowledge gap persists at the intersection of minimally traumatic surgical technology and host-related determinants of postoperative recovery, particularly in patients with coexisting CAD and DM [5,6]. This lack of evidence is important because the postoperative course in medically compromised patients cannot be assumed to mirror that of healthy individuals, even when the same minimally invasive device is used [4,5]. Clarifying this issue may help clinicians choose surgical approaches more appropriately for high-risk patients undergoing minor oral procedures [1,5].

Accordingly, the aim of this retrospective cohort study was to compare postoperative pain, swelling, and trismus after piezosurgery-assisted minor oral surgical procedures in patients with coronary artery disease and diabetes mellitus and in systemically healthy individuals. We hypothesized that piezosurgery would yield an acceptable postoperative course in the CAD+DM group, but that systemic disease might still influence the magnitude and temporal profile of postoperative morbidity compared with healthy controls. By focusing on real-world clinical outcomes in a medically vulnerable population, this study was designed to address a clinically relevant gap in the current literature on piezoelectric surgery in oral and maxillofacial practice.

## 2. Materials and Methods

### 2.1. Study Design and Ethical Approval

This retrospective cohort study was approved by the Ethics Committee of Ege University (Approval No: 21-4T/11). Given the retrospective design, the requirement for written informed consent was waived in accordance with institutional regulations and ethical guidelines.

### 2.2. Patient Selection

The medical records of 60 patients (21 females and 39 males) who underwent piezosurgery-assisted minor oral surgical procedures at the Department of Oral and Maxillofacial Surgery, Ege University, from March 2018 to March 2021 were retrospectively reviewed. Procedures included removal of impacted teeth, complicated tooth extraction, and lateral sinus lift surgery.

Patients were allocated to two groups based on systemic health status. CAD+DM group (n = 30): Patients diagnosed with coronary artery disease (CAD) and diabetes mellitus (DM). Healthy group (n = 30): Systemically healthy individuals without known cardiovascular or metabolic disorders.

All procedures included in the study were performed using piezosurgery.

### 2.3. Inclusion and Exclusion Criteria

Patients were eligible for inclusion if they:

1. Were classified as high-risk according to the National Cholesterol Education Program Adult Treatment Panel III (NCEP ATP III) criteria for CAD or CAD risk equivalents.
2. Had undergone at least one minor oral surgical procedure using piezosurgery.
3. Had complete postoperative follow-up records.

Oral hygiene was assessed preoperatively using the Greene–Vermillion Simplified Oral Hygiene Index (OHI-S). The OHI-S score ranges from 0 to 6, with higher scores indicating poorer oral hygiene. According to the original classification, an OHI-S score > 3.0 was considered indicative of poor oral hygiene, and patients exceeding this threshold were excluded from the study.

Patients were excluded if they had:

1. A history of acute myocardial infarction within the previous six months.
2. Planned cardiac surgery or angioplasty at the time of oral surgery.
3. Severe hypertension or uncontrolled diabetes mellitus.
4. Poor oral hygiene (OHI-S > 3.0).
5. Incomplete clinical records or missing postoperative evaluations.

### 2.4. Minor Oral Surgical Procedures

The procedures in the present study were considered minor oral surgical procedures within the scope of dentoalveolar and preimplant oral surgery and comprised impacted tooth removal, complicated tooth extraction, and lateral sinus floor elevation in the posterior maxilla [9,10].

### 2.5. Surgical Sites

Surgical sites were classified by anatomical region, and procedure types were classified by the surgical intervention performed. The interventions were performed in comparable anatomical regions in both groups. In the CAD+DM group, 15 procedures involved mandibular third molars, 7 involved maxillary third molars, and 8 involved the posterior maxilla for lateral sinus floor elevation. In the healthy group, 14 procedures involved mandibular third molars, 10 involved maxillary third molars, and 6 involved the posterior maxilla for lateral sinus floor elevation.

### 2.6. Distribution of Procedure Types

The procedures included impacted tooth removal, complicated tooth extraction, and lateral sinus lift surgery. The distribution of procedure types was as follows: in the CAD+DM group, impacted tooth removal (n = 14), complicated tooth extraction (n = 8), and lateral sinus lift surgery (n = 8); in the healthy group, impacted tooth removal (n = 14), complicated tooth extraction (n = 10), and lateral sinus lift surgery (n = 6). Procedure type was not included as a covariate because the number of patients in each procedural subgroup was insufficient to support reliable adjusted analyses. Therefore, the primary analyses were conducted on the overall study cohort rather than stratified by procedure type.

### 2.7. Surgical Procedure

All procedures were performed by a single experienced oral and maxillofacial surgeon under local anesthesia with 2% lidocaine with epinephrine (Jetokain^®^, Adeka, Turkey). After crestal and releasing incisions, full-thickness mucoperiosteal flaps were elevated. Osteotomy, osteoplasty, and alveoloplasty were performed using a piezoelectric surgical device (Piezon^®^ Master Surgery, EMS Electro Medical Systems S.A., Nyon, Switzerland). Surgical sites were closed with non-resorbable 4-0 sutures.

### 2.8. Clinical Evaluation

Postoperative pain was assessed with a 10-point visual analog scale (VAS), in which 0 indicated no pain and 10 the worst pain imaginable. The VAS is a widely used and validated instrument for measuring pain intensity in clinical and postoperative settings [11,12]. VAS scores were recorded at 6 h, 12 h, and on postoperative days 1, 2, 3, and 7.

Trismus was assessed by measuring the maximum interincisal distance (mm), a standard clinical measure of postoperative mouth-opening limitation in oral and maxillofacial surgery. Measurements were obtained preoperatively and on postoperative days 2 and 7 [2,3].

All postoperative swelling and trismus measurements were performed by a single experienced oral and maxillofacial surgeon who was blinded to the patients’ systemic health status. Because of the retrospective study design, formal inter- and intra-examiner reliability analyses were not conducted.

Facial swelling was assessed using the method described by Amin and Laskin [13]. Linear facial measurements were obtained from the tragus to the pogonion, from the tragus to the corner of the mouth, and from the lateral canthus of the eye to the angle of the mandible. Measurements were recorded preoperatively and on postoperative days 2 and 7.

### 2.9. Glycemic and Cardiovascular Variables

Patients in the CAD+DM group had a previously established diagnosis of diabetes mellitus and were receiving antidiabetic treatment at the time of surgery. Therefore, HbA1c values were recorded as descriptive indicators of perioperative glycemic control rather than used as diagnostic or inclusion criteria. Because glycemic control may improve substantially with ongoing pharmacological treatment, HbA1c values in patients with established diabetes may fall below the diagnostic threshold of 6.5% despite the persistence of the underlying disease. Accordingly, HbA1c was interpreted as a marker of current metabolic control rather than disease status in the present study. Information on the severity of coronary artery disease and on antiplatelet and/or anticoagulant therapy was recorded when available in the medical records. However, because these data were not consistently available for all patients, they were not included as covariates in the statistical analyses.

### 2.10. Statistical Analysis

Statistical analyses were conducted using IBM SPSS Statistics version 25.0 (IBM Corp., Armonk, NY, USA) and GraphPad Prism version 10. Data are presented as mean ± standard deviation (SD) or as median (interquartile range [IQR]), depending on the data distribution. Normality was assessed with the Shapiro–Wilk test. Swelling and trismus measurements were analyzed with repeated-measures analysis of variance (ANOVA) followed by Tukey’s multiple-comparisons test. Between-group comparisons were performed with two-way repeated-measures ANOVA. Because VAS scores were not normally distributed, within-group changes over time were evaluated with the Friedman test followed by Dunn’s multiple-comparisons test. Between-group comparisons at each time point were performed with the Mann–Whitney U test. A *p*-value < 0.05 was considered statistically significant.

#### Statistical Considerations

This study was designed as an exploratory retrospective cohort analysis of all consecutive eligible cases identified in the institutional archive during the study period. Because the final sample included all consecutive patients who met the inclusion criteria and had complete follow-up records, no a priori sample size calculation was performed. Therefore, the findings should be interpreted in the context of the exploratory design and the relatively limited sample size.

## 3. Results

### 3.1. Patient Characteristics

There was no significant difference in age between the CAD+DM and healthy groups (46.8 ± 14.9 vs. 40.7 ± 17.0 years, *p* = 0.133).

### 3.2. Swelling Measurements

In the healthy group, swelling measurements changed significantly over time (repeated-measures ANOVA, F(1.272, 36.90) = 13.05, *p* = 0.0004). Post hoc Tukey analysis demonstrated that swelling was significantly higher on postoperative day 2 (POD2) than during the preoperative period (13.77 ± 1.05 mm vs. 14.13 ± 1.29 mm, *p* = 0.0018). Swelling decreased significantly from POD2 to postoperative day 7 (POD7) (14.13 ± 1.29 mm vs. 13.78 ± 1.00 mm, *p* = 0.0024). No significant difference was observed between preoperative and POD7 measurements (13.77 ± 1.05 mm vs. 13.78 ± 1.00 mm, *p* = 0.9391).

In the CAD+DM group, swelling measurements also changed significantly over time (repeated-measures ANOVA, F(1.765, 51.20) = 91.89, *p* < 0.0001). Swelling increased significantly from the preoperative period to POD2 (14.82 ± 1.72 mm vs. 15.66 ± 1.75 mm, *p* < 0.0001) and decreased significantly from POD2 to POD7 (15.66 ± 1.75 mm vs. 15.00 ± 1.78 mm, *p* < 0.0001). No significant difference was found between preoperative and POD7 measurements (14.82 ± 1.72 mm vs. 15.00 ± 1.78 mm, *p* = 0.0744).

**Figure 1:**
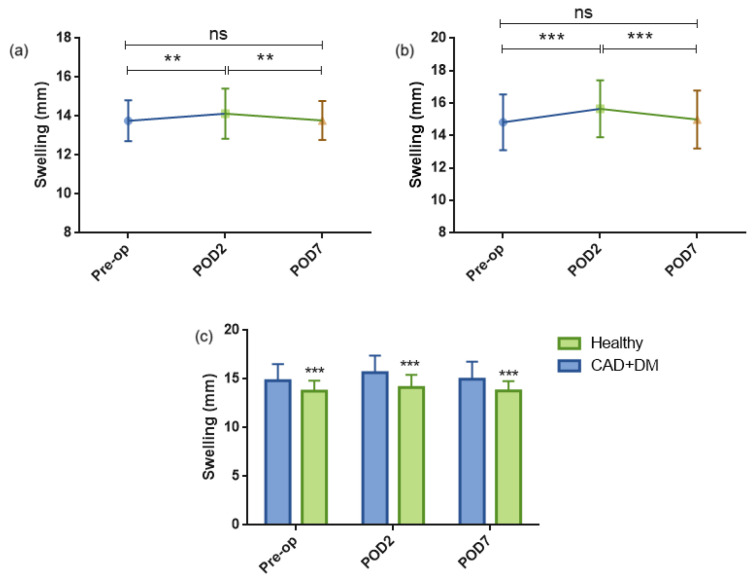
Swelling measurements in the healthy and CAD+DM groups. (**a**) Within-group comparison in the healthy group. (**b**) Within-group comparison in the CAD+DM group. (**c**) Between-group comparison at each time point. Repeated-measures one-way ANOVA revealed significant time-dependent changes in both groups, whereas two-way repeated-measures ANOVA demonstrated significant effects of time, group, and time × group interaction (all *p* < 0.001). Data are presented as mean ± SD. ** *p* < 0.01, *** *p* < 0.001; ns, not significant. POD2, postoperative day 2; POD7, postoperative day 7. ns: not significant.

Two-way repeated-measures ANOVA demonstrated significant effects of time (F(2,58) = 76.86, *p* < 0.0001), group (F(1,29) = 70.12, *p* < 0.0001), and time × group interaction (F(2,58) = 11.24, *p* < 0.0001). Swelling values were significantly higher in the CAD+DM group than in the healthy group at all evaluation time points (all *p* < 0.0001) (Figure 1, Table 1).

### 3.3. Trismus Measurements

In the healthy group, trismus measurements changed significantly over time (repeated-measures ANOVA, F(1.626, 43.89) = 351.4, *p* < 0.0001). Post hoc Tukey analysis demonstrated that maximum mouth opening was significantly reduced on postoperative day 2 (POD2) compared with the preoperative period (48.38 ± 3.49 mm vs. 32.30 ± 3.19 mm, *p* < 0.0001). Mouth opening increased significantly from POD2 to postoperative day 7 (POD7) (32.30 ± 3.19 mm vs. 44.45 ± 2.80 mm, *p* < 0.0001). Although POD7 values remained significantly lower than preoperative values (48.38 ± 3.49 mm vs. 44.45 ± 2.80 mm, *p* < 0.0001), substantial recovery was observed by postoperative day 7.

In the CAD+DM group, trismus measurements also changed significantly over time (repeated-measures ANOVA, F(1.945, 56.40) = 146.0, *p* < 0.0001). Maximum mouth opening decreased significantly from the preoperative period to POD2 (45.04 ± 5.30 mm vs. 35.87 ± 5.89 mm, *p* < 0.0001) and increased significantly from POD2 to POD7 (35.87 ± 5.89 mm vs. 44.01 ± 5.74 mm, *p* < 0.0001). No significant difference was observed between preoperative and POD7 measurements (45.04 ± 5.30 mm vs. 44.01 ± 5.74 mm, *p* = 0.1541).

**Figure 2:**
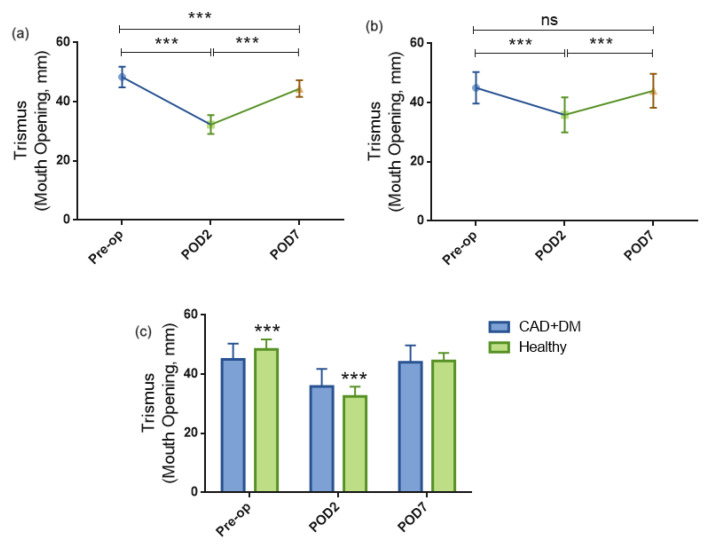
Changes in trismus (maximum mouth opening) measurements over time in the CAD+DM and healthy groups. (**a**) Within-group comparison in the healthy group. (**b**) Within-group comparison in the CAD+DM group. (**c**) Between-group comparison at each evaluation time point. Repeated-measures one-way ANOVA demonstrated significant time-dependent changes in both groups, whereas two-way repeated-measures ANOVA revealed significant effects of time and time × group interaction (both *p* < 0.001). Data are presented as mean ± SD. *** *p* < 0.001; ns, not significant. POD2, postoperative day 2; POD7, postoperative day 7. ns: not significant.

Two-way repeated-measures ANOVA demonstrated a significant effect of time (F(2,58) = 556.8, *p* < 0.0001) and a significant time × group interaction (F(2,58) = 28.03, *p* < 0.0001), whereas the overall group effect was not significant (F(1,29) = 0.0215, *p* = 0.8844). Between-group comparisons revealed significantly greater mouth opening in the healthy group during the preoperative period (48.38 ± 3.49 mm vs. 45.04 ± 5.30 mm, *p* < 0.0001) and significantly lower mouth opening in the healthy group on POD2 (32.30 ± 3.19 mm vs. 35.87 ± 5.89 mm, *p* < 0.0001). However, no significant difference was observed between groups on POD7 (44.45 ± 2.80 mm vs. 44.01 ± 5.74 mm, *p* = 0.7729) (Figure 2, Table 2).

### 3.4. VAS Scores

VAS scores were not normally distributed; therefore, non-parametric analyses were used. In the CAD+DM group, VAS scores changed significantly over time (Friedman statistic = 142.0, *p* < 0.0001). Dunn’s multiple-comparisons test showed that VAS scores were significantly lower from postoperative day 2 onward compared with the early postoperative measurements.

In the healthy group, VAS scores also changed significantly over time (Friedman statistic = 123.3, *p* < 0.0001). Pain scores decreased progressively after 12 h, with significantly lower values observed from postoperative day 2 to day 7. Overall, VAS scores were low in both groups throughout follow-up, but postoperative pain persisted longer in the healthy group than in the CAD+DM group.

Between-group comparisons performed using the Mann–Whitney U test demonstrated significantly lower pain scores in the CAD+DM group at 6 h (*p* < 0.001), postoperative day 1 (*p* < 0.001), postoperative day 2 (*p* < 0.001), postoperative day 3 (*p* < 0.001), and postoperative day 7 (*p* < 0.001). No significant difference was observed between groups at 12 h (*p* = 0.633).

**Figure 3:**
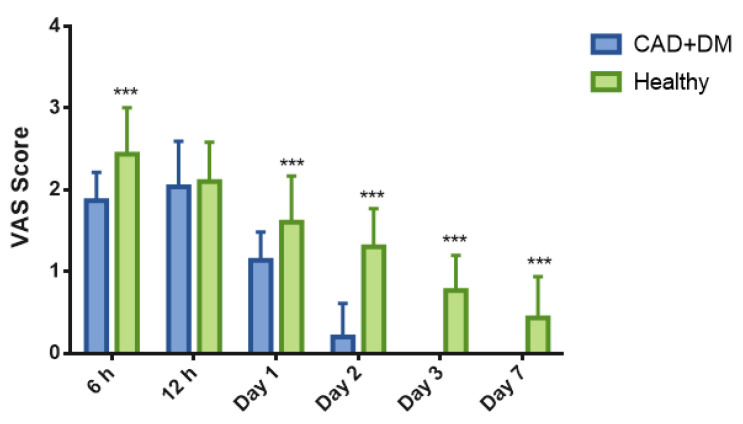
Comparison of postoperative VAS scores between the CAD+DM and healthy groups. VAS scores were assessed at 6 h, 12 h, postoperative day 1 (Day 1), day 2 (Day 2), day 3 (Day 3), and day 7 (Day 7). Pain scores decreased progressively in both groups throughout the postoperative period. Between-group comparisons were performed using the Mann–Whitney U test. Data are presented as median (IQR). *** *p* < 0.001 versus the CAD+DM group.

Overall, postoperative pain decreased over time in both groups; however, pain resolution occurred earlier in the CAD+DM group, whereas pain persisted longer in the healthy group (Figure 3, Table 3).

## 4. Discussion

In this retrospective cohort study, piezosurgery-assisted minor oral surgery was associated with favorable early postoperative recovery in both systemically healthy individuals and patients with concomitant coronary artery disease and diabetes mellitus (CAD+DM). Although postoperative pain remained generally low and trismus improved progressively in both groups, patients with CAD+DM consistently exhibited greater postoperative swelling throughout the follow-up period. These findings suggest that the minimally invasive nature of piezosurgery may support satisfactory early postoperative recovery even in medically compromised patients. However, the presence of combined cardiovascular and metabolic disease appears to influence the magnitude of the postoperative inflammatory response, indicating that postoperative recovery is determined not only by the surgical technique but also by the patient’s underlying systemic condition [5,14].

The swelling pattern observed in the present study aligns with previous reports indicating that postoperative edema peaks during the early inflammatory phase and gradually subsides during the first postoperative week after piezosurgery-assisted oral surgery [3,15,16]. However, the persistently greater swelling in the CAD+DM group suggests that the reduction in surgical trauma achieved by piezoelectric osteotomy may not fully offset the adverse effects of systemic metabolic and vascular disease on postoperative tissue repair [5,14]. These alterations include endothelial dysfunction, oxidative stress, altered macrophage behavior, persistent low-grade inflammation, and impaired progression from inflammatory to reparative healing phases, all of which may contribute to more pronounced postoperative edema after surgical injury [5,17,18]. Although HbA1c values were available as descriptive indicators of glycemic control, comprehensive metabolic assessment, cardiovascular disease severity, and inflammatory biomarkers were not uniformly available in this retrospective cohort. Therefore, these proposed mechanisms should be regarded as biologically plausible interpretations rather than directly demonstrated explanations.

Trismus followed a typical postoperative trajectory in both groups, with the greatest limitation in mouth opening on postoperative day 2 and marked recovery by postoperative day 7. Previous oral surgery studies using piezosurgery have likewise reported transient early restriction in mouth opening, with progressive improvement during the first postoperative week [3,15,19]. Importantly, despite differences in baseline values and early postoperative change, no between-group difference was observed on postoperative day 7 in the present study. This finding suggests that short-term functional recovery of jaw opening may be broadly comparable between groups, even when the CAD+DM group exhibits greater facial swelling. Because trismus may reflect multiple postoperative factors, including muscular guarding, periosteal irritation, local edema, and procedure-related tissue manipulation, it does not necessarily parallel facial swelling in a linear manner after oral surgery [3,15]. Nevertheless, this interpretation should be considered with caution because the present cohort included heterogeneous procedures and did not include procedure-specific adjusted analyses or adjustment for other potential confounders.

The most intriguing finding of the present study was that VAS scores were lower in the CAD+DM group at most postoperative time points, despite greater swelling, a result that seems counterintuitive if pain is viewed solely as a consequence of tissue inflammation. A plausible explanation is diabetic peripheral neuropathy, which may reduce nociceptive input, impair sensory perception, and diminish awareness of tissue injury, particularly in patients with longstanding diabetes mellitus [6,20,21,22]. However, this interpretation remains speculative because neuropathy status, diabetes duration, and postoperative analgesic consumption were not systematically documented in the retrospective records. Although HbA1c values were available as descriptive indicators of glycemic control, detailed metabolic characterization and standardized neuropathy assessment were not available. In addition, diabetic neuropathy is a heterogeneous condition and may manifest as sensory loss rather than pain, so lower self-reported VAS scores cannot be assumed to reflect a single mechanism across all patients [6,21,23,24].

Furthermore, chronic cardiovascular medication use and other unmeasured clinical variables may have influenced pain perception or reporting; however, the retrospective design of the present study did not allow these potential effects to be evaluated independently [24,25]. Therefore, the lower pain scores observed in the CAD+DM group should be interpreted as an interesting but non-definitive finding rather than evidence of superior biological recovery. Prospective studies incorporating standardized assessments of diabetic neuropathy, diabetes duration, medication profiles, and postoperative analgesic consumption are warranted to clarify the mechanisms underlying postoperative pain perception in medically compromised patients [21,24].

Despite these between-group differences, postoperative pain remained low in both groups throughout follow-up. This pattern aligns with the proposed advantages of piezosurgery, including reduced collateral soft-tissue trauma, lower mechanical stress during osteotomy, and improved surgical precision compared with conventional rotary instrumentation [2,15,19,26]. Nevertheless, the present findings should be interpreted in light of the study’s limitations and suggest that piezosurgery may be a useful surgical approach for medically compromised patients, although the influence of underlying systemic disease on postoperative recovery should not be overlooked [1,5].

## 5. Limitations

Several limitations of this study should be acknowledged. First, the retrospective single-center design and relatively limited sample size restrict causal inference, so the findings should be interpreted as exploratory. Second, the cohort included various minor oral surgical procedures, but the number of patients in each procedural subgroup was insufficient to permit reliable procedure-specific adjusted analyses. Third, although HbA1c values were available as descriptive indicators of glycemic control, important clinical variables—including neuropathy status, diabetes duration, cardiovascular disease severity, perioperative analgesic use, inflammatory biomarkers, and detailed medication profiles—were not consistently available for all patients and therefore could not be incorporated into the adjusted analyses. Finally, because the study did not include a conventional rotary osteotomy control group, the present findings should not be interpreted as evidence of the superiority of piezosurgery over other surgical techniques.

## 6. Conclusions

Within the limitations of this single-center retrospective cohort study, piezosurgery-assisted minor oral surgery was associated with a favorable early postoperative course in both CAD+DM patients and systemically healthy individuals. Although patients with CAD+DM exhibited greater postoperative swelling, recovery of mouth opening was comparable between groups by postoperative day 7, and overall postoperative pain remained low. These findings should be considered exploratory because of the retrospective design, relatively small sample size, heterogeneous procedure types, and incomplete availability of detailed metabolic, neuropathy-related, medication, and cardiovascular variables. Larger prospective studies incorporating procedure-specific analyses, standardized metabolic and neuropathy assessments, and conventional osteotomy control groups are warranted to confirm these findings and further clarify postoperative recovery in medically compromised patients.

## Figures and Tables

**Table 1:** Swelling measurements in the CAD+DM and healthy groups.

**Time point**	**CAD+DM (n = 30) Mean ± SD (mm)**	**Healthy (n = 30) Mean ± SD (mm)**	**Between-group *p* value**
Preoperative	14.82 ± 1.72	13.77 ± 1.05	<0.0001
POD2	15.66 ± 1.75	14.13 ± 1.29	<0.0001
POD7	15.00 ± 1.78	13.78 ± 1.00	<0.0001
**Within-group comparisons**			
**Comparison**	**CAD+DM *p* value**		**Healthy *p* value**
Pre-op vs. POD2	<0.0001		0.0018
Pre-op vs. POD7	0.0744		0.9391
POD2 vs. POD7	<0.0001		0.0024

Data are presented as mean ± SD. Between-group comparisons were performed using two-way repeated-measures ANOVA with Bonferroni post hoc testing. Within-group comparisons were performed using repeated-measures ANOVA followed by Tukey’s multiple-comparisons test. POD2, postoperative day 2; POD7, postoperative day 7.

**Table 2:** Trismus (maximum mouth opening) measurements in the CAD+DM and healthy groups.

**Time point**	**CAD+DM (n = 30) Mean ± SD (mm)**	**Healthy (n = 28) Mean ± SD (mm)**	**Between-group *p* value**
Preoperative	45.04 ± 5.30	48.38 ± 3.49	<0.0001
POD2	35.87 ± 5.89	32.30 ± 3.19	<0.0001
POD7	44.01 ± 5.74	44.45 ± 2.80	0.7729
**Within-group comparisons**			
**Comparison**	**CAD+DM *p* value**		**Healthy *p* value**
Pre-op vs POD2	<0.0001		<0.0001
Pre-op vs POD7	0.1541		<0.0001
POD2 vs POD7	<0.0001		<0.0001

Data are presented as mean ± SD. Between-group comparisons were performed using two-way repeated-measures ANOVA with Bonferroni post hoc testing. Within-group comparisons were performed using repeated-measures ANOVA followed by Tukey’s multiple-comparisons test. POD2, postoperative day 2; POD7, postoperative day 7.

**Table 3:** VAS scores in the CAD+DM and Healthy groups.

**Time**	**CAD+DM (n = 30) Median (IQR)**	**Healthy (n = 30) Median (IQR)**	Between-group *p* value *
6 h	2.00 (2.00–2.00)	2.00 (2.00–3.00)	<0.001
12 h	2.00 (2.00–2.00)	2.00 (2.00–2.00)	0.633
Day 1	1.00 (1.00–1.00)	2.00 (1.00–2.00)	<0.001
Day 2	0.00 (0.00–0.00)	1.00 (1.00–2.00)	<0.001
Day 3	0.00 (0.00–0.00)	1.00 (0.75–1.00)	<0.001
Day 7	0.00 (0.00–0.00)	0.00 (0.00–1.00)	<0.001
**Within-group Friedman analysis**			
**Group**	**Friedman statistic**		***p* value**
CAD+DM	142.0		<0.0001
Healthy	123.3		<0.0001

* Between-group comparisons were performed using the Mann–Whitney U test.

## Data Availability

Data are available on request from the corresponding author due to privacy/ethical reasons.
